# Choroidal microcirculation in pediatric craniopharyngioma: postoperative alterations in choroidal thickness

**DOI:** 10.3389/fped.2026.1826785

**Published:** 2026-07-27

**Authors:** Klaudia Rakusiewicz-Krasnodębska, Agnieszka Bogusz-Wójcik, Elżbieta Moszczyńska, Maciej Jaworski, Paweł Kowalczyk, Wojciech Hautz

**Affiliations:** 1 Department of Pediatric Ophthalmology, Children’s Memorial Health Institute, Warsaw, Poland; 2 Department of Pediatric Endocrinology and Diabetology, Children’s Memorial Health Institute, Warsaw, Poland; 3 Department of Clinical Biochemistry, Children’s Memorial Health Institute, Warsaw, Poland; 4 Department of Pediatric Neurosurgery, Children’s Memorial Health Institute, Warsaw, Poland

**Keywords:** choroidal thickness, compressive optic neuropathy, craniopharyngioma, optic chiasma, optical coherence tomography

## Abstract

**Purpose:**

To evaluate choroidal microcirculation using choroidal thickness (CTh) in pediatric patients with surgically treated adamantinomatous craniopharyngioma (CP) and to identify clinical and tumor-related factors associated with CTh.

**Methods:**

This cross-sectional, prospective, observational study included 60 eyes of 31 children with histopathologically confirmed CP and 78 eyes of 39 age- and sex-matched healthy controls. Comprehensive ophthalmological examination and spectral-domain optical coherence tomography (SD-OCT) were performed in all participants. CTh was manually measured at five locations: subfoveal (SFCTh), 1500 μm superior, inferior, nasal, and temporal to the fovea in both eyes. Tumor characteristics and demographic data were collected and analyzed for associations with CTh.

**Results:**

CTh was significantly thinner in the CP group compared to controls at all locations except 1500 μm superior to the fovea. The mean subfoveal CTh was 333.13 μm in the CP group and 370.13 μm in the control group (*p* < 0.05). The most pronounced thinning was observed nasally (255.93 vs. 335.06 μm, *p* < 0.05). At the temporal location, mean CTh was 317.62 μm in the CP group and 360.62 μm in controls (*p* < 0.05). Inferiorly, it measured 318.88 μm in the CP group vs. 346.04 μm in controls (*p* < 0.05). Superior CTh was 340.23 μm in the CP group and 358.47 μm in controls, with no statistically significant difference (*p* = 0.08). In the study group, the thickest CTh was found superiorly, while in the control group, it was SFCTh. In both groups, the thinnest CTh was observed nasally. Children born in the city had a significantly thicker SFCTh than those born outside the city (*p* < 0.005). Cavernous sinus infiltration was associated with increased nasal CTh and temporal CTh (*p* < 0.005). No association was found between CTh and tumor location, tumor volume, tumor diameter, morphology, third ventricle invasion, hydrocephalus, Rosenthal fibers or ventriculoperitoneal shunt. Age at examination and gender did not affect CTh.

**Conclusion:**

Pediatric patients with CP show significantly reduced CTh in multiple locations. Place of birth, and specific tumor characteristics such as cavernous sinus infiltration were influenced by CTh. These findings highlight the complex interplay between tumor characteristics and ocular microvascular changes in pediatric patients with CP.

## Introduction

Craniopharyngioma (CP) is a rare, histologically benign, and slow-growing yet clinically challenging brain tumor that predominantly affects the pediatric population, most commonly between the ages of 5 and 15, and again in adults aged 45 to 60, showing a bimodal age distribution. Originating in the sella turcica and suprasellar region, it often compresses adjacent neurovascular structures, particularly the optic chiasm, optic nerves, pituitary gland, and cerebral arteries, commonly referred to as the circle of Willis, leading to visual disturbances, endocrine dysfunction, and hypothalamic disorders (1–5). Surgical resection remains the cornerstone of treatment, although it is associated with a high risk of complications, including panhypopituitarism and visual loss due to the tumor's proximity to the visual pathways, as well as hypothalamic obesity, sleep and thermoregulation disturbances, cognitive impairment, and behavioral or psychosocial difficulties that may significantly impact long-term quality of life (1, 2).

Visual prognosis is one of the key concerns in pediatric CP and strongly influences quality of life (3–5). Visual outcomes depend not only on direct tumor compression but also on secondary mechanisms including disrupted axoplasmic flow, altered myelin integrity, and mitochondrial dysfunction (5, 6). These processes are likely related to the local microcirculatory environment, where hypoperfusion and ischemia could potentially contribute to neurodegenerative changes (7, 8). Moreover, tumor mass effect and growth may disturb microcirculation both locally and in distant areas (9).

The choroid, one of the most vascularized tissues in the body, plays a critical role in maintaining the metabolic and structural integrity of the outer retina. Disturbances in choroidal circulation may lead to photoreceptor ischemia, oxidative stress, and vision loss (10–12). Importantly, choroidal perfusion may be influenced by intracranial tumors, neurosurgical interventions, and intracranial pressure changes (13–15).

Historically, choroidal imaging was limited by low-resolution techniques. The advent of optical coherence tomography (OCT), particularly spectral-domain OCT (SD-OCT), enabled high-resolution, non-invasive assessment of choroidal thickness (CTh), now recognized as a quantitative biomarker of vascular integrity and perfusion (16–18). Alterations in CTh have been reported across a wide range of ocular and systemic diseases, including glaucoma, age-related macular degeneration, uveitis, myopia, and cardiovascular or metabolic disorders (18–28).

Despite the clinical importance of visual outcomes in pediatric CP, data on choroidal structure in this population remain scarce. Considering the close anatomical relationship between the suprasellar region and the visual system, evaluating ocular microvasculature may provide additional information on CP-related pathophysiology. Choroidal thickness (CTh) could potentially reflect local or systemic vascular changes, though its role as a biomarker of disease burden remains to be established.

This prospective, cross-sectional study aimed to evaluate CTh in pediatric patients with histopathologically confirmed adamantinomatous CP following neurosurgical treatment, and to compare these findings with age- and sex-matched healthy controls. Furthermore, we investigated associations between CTh and demographic or tumor-related factors, such as the presence of calcifications, Rosenthal fibers, cavernous sinus infiltration, and tumor morphology. To our knowledge, this is the first study to present data on CTh in pediatric patients with CP.

## Materials and methods

This cross-sectional, prospective, observational study was conducted at the Departments of Endocrinology and Ophthalmology at the Children's Memorial Health Institute in Warsaw, Poland, between September 2021 and April 2025. The main inclusion criteria were age below 18 years and the presence of a sellar mass on pituitary magnetic resonance imaging (MRI), which was surgically treated and histopathologically confirmed as adamantinomatous CP. The study complied with the principles of the Declaration of Helsinki and received approval from the Bioethics Committee of the Children's Memorial Health Institute in Warsaw on 19 June 2024 (resolution number 21/KBE/2024). Participants aged over 13 and their guardians, as well as the legal guardians of those under 13, were provided with detailed information about the study's purpose and potential risks, and gave written informed consent to participate.

A total of 60 eyes from 32 children with CP (17 males, 15 females; mean age: 10.28 ± 4.25 years; range: 4–17 years) who had undergone neurosurgical treatment and were followed at the Department of Endocrinology and the Department of Ophthalmology at the Children's Memorial Health Institute were enrolled in the study. The inclusion criterion for the study group was histopathological confirmation of adamantinomatous CP in postoperative tissue samples. The control group consisted of 78 eyes from 39 healthy children without CP or other systemic or ocular diseases, with a sex distribution (16 M/32 F) and age distribution (mean age 10.93 ± 3.26; range 4–16 years) similar to those of the study group. The analysis was conducted at the eye level (*n* = 60/78) rather than the patient level (*n* = 32/39), with each eye treated as an independent observational unit due to asymmetric disease involvement, as commonly adopted in similar OCT studies.

**Figure 1:**
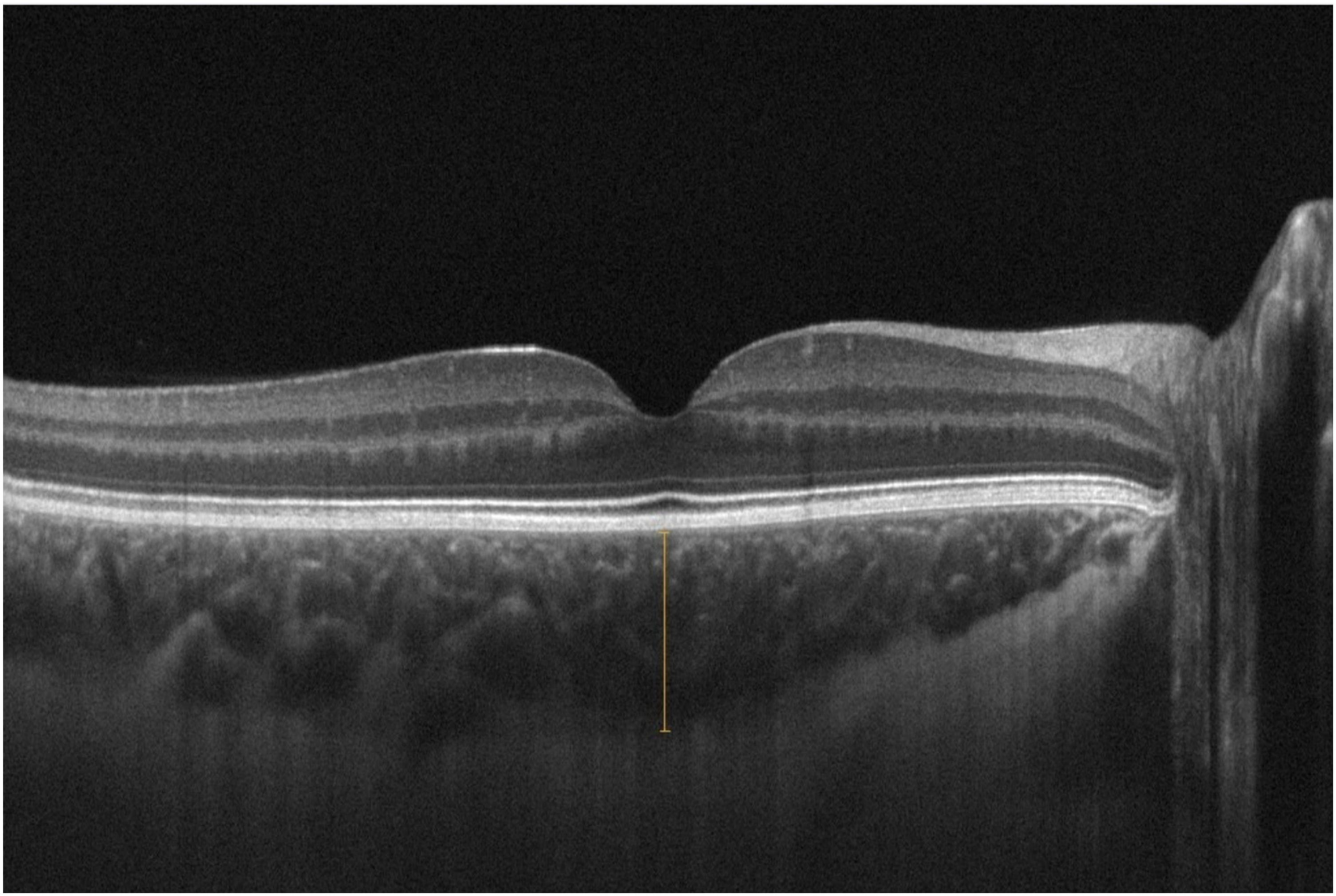
Transverse scan through the retina and choroid. The orange line indicates the measurement of choroidal thickness, extending from the hyperreflective line corresponding to the outer border of the retinal pigment epithelium (RPE) to the hyperreflective line corresponding to the inner scleral boundary.

Exclusion criteria for both groups included a history of ocular surgery or trauma; coexisting ophthalmic conditions such as glaucoma, uveitis, optic nerve diseases, hereditary retinal dystrophies, retinal or macular diseases, vitreoretinal disorders, and significant corneal or lens opacities. Patients with significant refractive errors (spherical > ±3.0 D, cylindrical > ±3.0 D), low-quality OCT scans, or systemic comorbidities known to affect CTh were also excluded. A total of 16 eyes from 10 patients were excluded from the study group owing to insufficient image quality, which did not allow for reliable CTh measurements. Additionally, individuals with previously diagnosed and treated systemic conditions such as diabetes mellitus, isolated hypertension, renal disease, neurological disorders, or a history of prematurity were not eligible for inclusion. All patients in the study group underwent comprehensive evaluations both before and after surgery. Preoperative assessments included gender, place of birth (city/village), and age at the time of CP diagnosis. Tumor characteristics were also analyzed, including location (suprasellar, intrasellar, or both supra and infrasellar), tumor volume, maximum diameter, morphology (solid, cystic, or mixed), presence of calcifications, presence of Rosenthal fibers in histopathological assessment (indicative of glial tissue reaction to the tumor), invasion of the third ventricle, cavernous sinus infiltration, hydrocephalus, and the presence of a ventriculoperitoneal shunt. Following surgery, all patients developed hypopituitarism, and hormone replacement therapy was initiated and monitored. The detailed clinical characteristics of the study group are summarized in Table 1. All patients in both the study and control groups underwent a comprehensive ophthalmological examination, including best-corrected visual acuity (BCVA) assessment, slit-lamp biomicroscopy of the anterior segment, and dilated fundus examination following pupil dilation with 1% tropicamide. SD-OCT was performed in all participants using a commercially available RTVue XR Avanti OCT system (Optovue, Fremont, California, USA). A crossline scan was used to obtain high-quality images of the retina and choroid. CTh was measured manually using the caliper tool in the SD-OCT software. CTh was defined as the vertical distance between the hyperreflective line corresponding to the outer border of the retinal pigment epithelium (RPE) and the hyperreflective line corresponding to the inner scleral border (Figure 1). The examinations included in the analysis were performed between 1.5 and 5 years after surgery, a period during which choroidal thickness was considered stable and no longer showed progressive deterioration. The mean interval between surgery and the OCT examination was approximately 3 years and 8 months.

**Table 1:** Demographic and eye data for CP patients and controls.

Group	Patients (n)	Eyes (n)	Male	Female	Mean age (years ± SD)	Age range (years)
CP group	32	60	17	15	10.28 ± 4.25	4–17
Control group	39	78	16	23	10.93 ± 3.26	4–16

Demographic and ocular characteristics of pediatric patients with craniopharyngioma (CP) following neurosurgical treatment and age- and sex-matched healthy controls. Data are presented as number of patients/eyes, mean ± standard deviation (SD) for age, and range.

**Figure 2:**
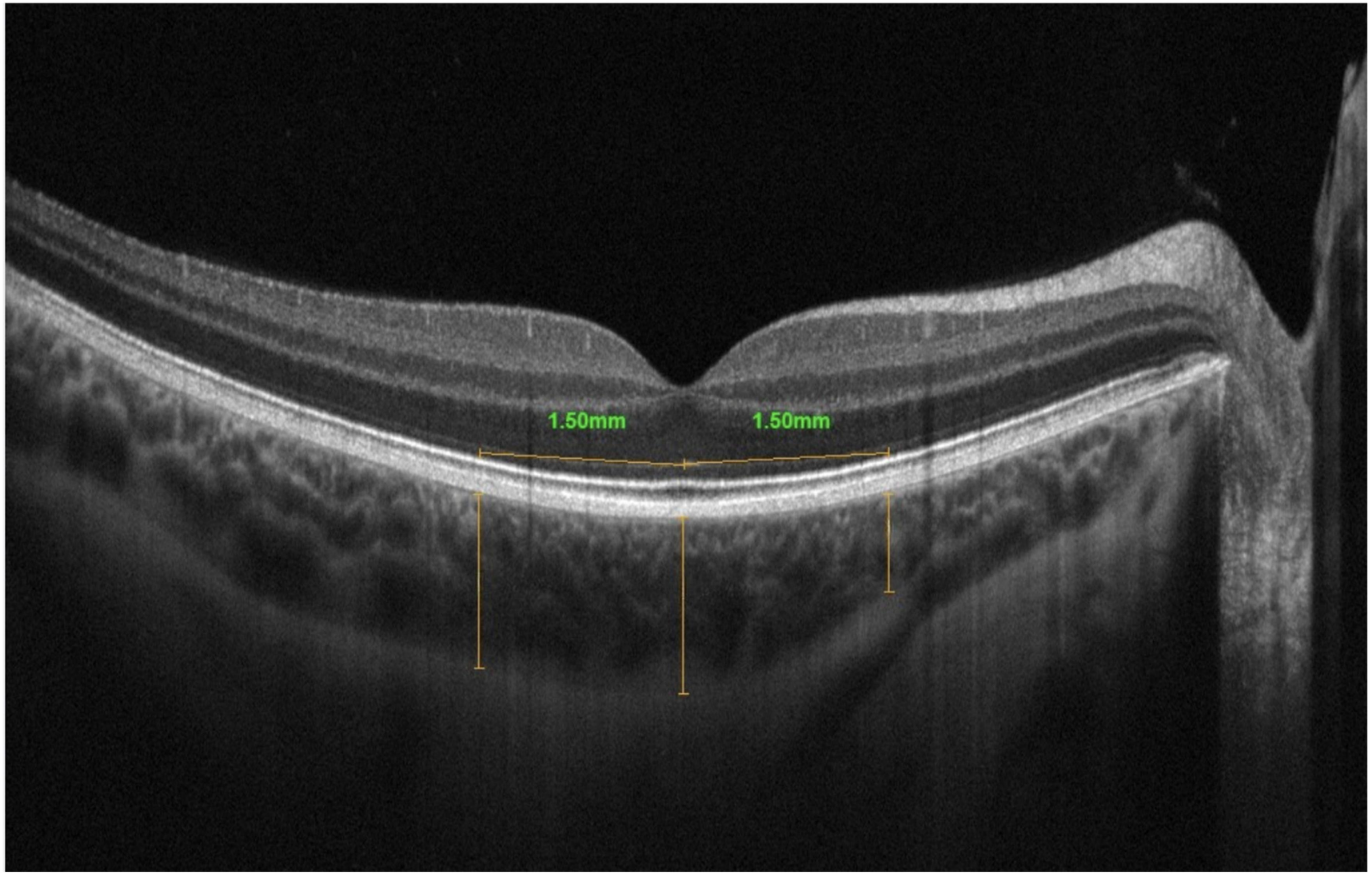
Manual measurement of choroidal thickness performed by The Examiner using spectral-domain optical coherence tomography. Measurements were taken subfoveally, as well as 1500 µm nasally and temporally from the fovea.

Measurements were performed by two independent investigators (KRK and ABW), and the average of their readings was used for analysis. CTh was measured in the subfoveal region defined as the lowest point on the retinal tomogram (SFCTh) and at 1500 μm superior, inferior, nasal, and temporal to the fovea (Figure 2). The measurement point located 1500 µm from the foveal center was selected based on previous choroidal imaging studies, as it provides a representative assessment of the parafoveal choroid and is sensitive to regional changes in choroidal vascular perfusion while avoiding the foveal avascular zone.

To minimize the effect of diurnal variation in choroidal thickness, all measurements were taken at the same time of day, between 11:00 a.m. and 1:00 p.m. Data from both eyes were included in the analysis.

Statistical Analysis:

Statistical analyses were performed using Statistica software, version 10 (StatSoft Inc., Tulsa, OK, USA). To evaluate the normality of variable distributions, the Shapiro–Wilk test was employed. Categorical variables were summarized as frequencies and percentages, whereas continuous variables were expressed as medians accompanied by interquartile ranges (IQR) and the full range (minimum to maximum values). Non-normally distributed variables were analysed after Box-Cox transformation. Associations between CTh and studied variables were evaluated using mixed-effects models. To account for multiple comparisons, p-values were adjusted using the Holm–Bonferroni correction according to the measured variables, groups of factors, and type of analysis (29). Comparisons of CTh between the right and left eyes were performed using the paired Student's t-test. Statistical significance was defined as a p-value below 0.05.

## Results

**Figure 3:**
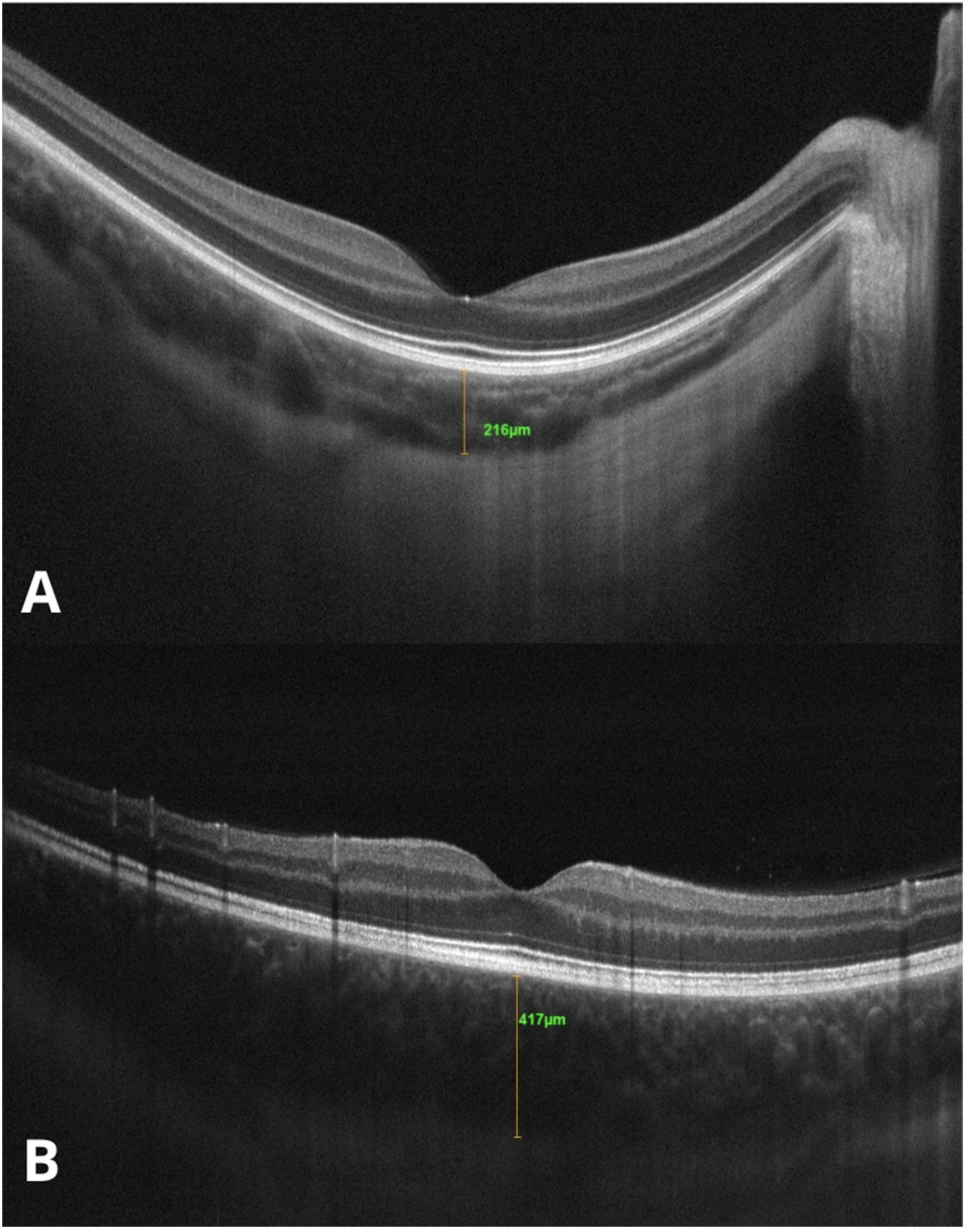
Measurement of choroidal thickness in the subfoveal region (SFCTh) in the study group with craniopharyngioma **(A)** and the control group **(B).**

Data were collected for 60 eyes of 32 patients (17M/15F) with neurosurgery treatment due to CP and 78 eyes of 39 patients (16 M/ 23F) in the control group. The mean age was 10.28 ± 4.25 years (range 4–17 years) for patients with CP and 10.93 ± 3.26 years (range 4–16 years) for controls. Demographic data, including age and gender, were comparable in both groups (Table 1). The CTh in all locations included in the analysis, except superior CTh, was statistically significantly thinner in patients with CP compared to the control group, and the results are presented in Table 2. Mean CTh in the group with CP compared to the control group was (333.13 vs. 370.13 μm, *p* <0.05) at the SFCTh (Figure 3), (255.93 vs. 335.06 μm, *p* < 0.05) 1500 μm nasal to the fovea, (317.62 vs. 360.62 μm, *p* < 0.05) 1500 μm temporal to the fovea, (340.23 vs. 358.47 μm, *p* = 0.08) 1500 μm superior to the fovea, (318.88 vs. 346.04 μm, *p* < 0.05) 1500 μm inferior to the fovea. The average difference between the groups ranged from 18.99 to 79.13 μm at individual locations. In the CP group, CTh was the thickest 1500 μm superiorly from the fovea location (340.23 μm), but in the control group, it was SFCTh (370.13 μm), and the thinnest was 1500 μm nasally from the fovea (255.93 vs. 335.06 μm) in both groups.

**Table 2:** Choroidal thickness at individual locations in CP patients and control subjects.

Variable	Study Group	M	Q1	Q3	Range	*p*
SFCTh	CP group	327,5	290,0	383,0	211–469	<0,05
	Control group	366,0	328,0	415,0	250–532	
Nasal CTh	CP group	243,0	204,5	299,5	155–407	<0.05
	Control group	327,5	299,0	361,0	235–469	
Temporal CTh	CP group	316,5	271,0	371,5	158–447	<0.05
	Control group	357,5	324,0	384,0	262–528	
Superior CTh	CP group	336,0	299,5	384,5	226–461	0,08
	Control group	352,5	321	398,0	255–469	
Inferior CThik	CP group	329,0	278,0	368,5	179–429	<0.05
	Control group	341,0	313,0	373,0	250–538	

M, median; Q1 lower quartile represents the 25th percentile of the data; Q3 upper quartile corresponds to the 75th percentile; p-value: statistical significance value, *SFCTh* subfoveal choroidal thickness, *CTh* choroidal thickness.

**Figure 4:**
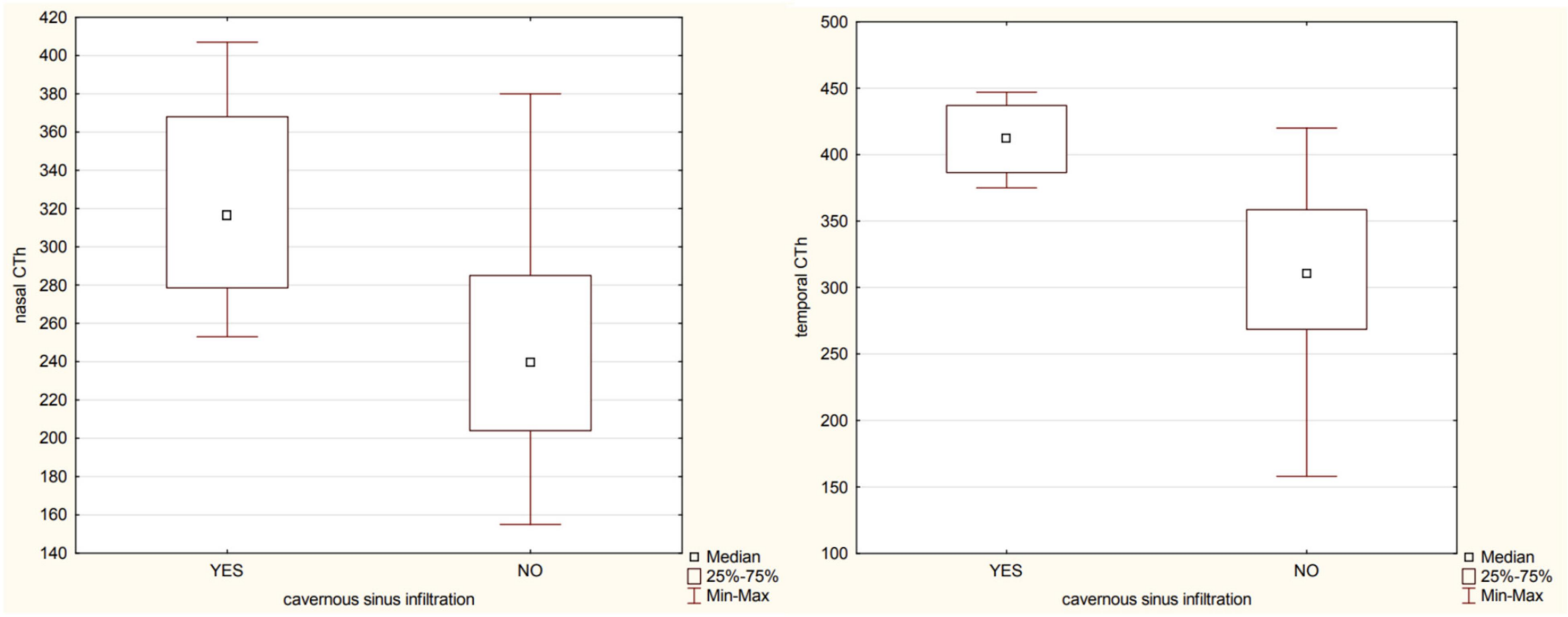
Graph showing the effect of presence of cavernous sinus infiltration on nasal and temporal CTh.

Gender was not significantly associated with CTh, although females tended to exhibit greater temporal CTh than males in both the CP and control groups (*p* = 0.057). Place of birth influenced SFCTh; patients born in urban areas in the city had significantly thicker SFCTh (*p* < 0.005). Age at the time of examination did not considerably affect CTh at any location. The presence of cavernous sinus infiltration was associated with greater CTh in the nasal and temporal regions (*p* < 0.005) (Figure 4).

In contrast, tumour location (suprasellar, infrasellar, or both), tumour volume, maximum tumour diameter, tumour morphology (solid, cystic, or mixed), involvement of the third ventricle, presence of hydrocephalus, tumor calcification, Rosenthal fibers or ventriculoperitoneal shunt placement had no significant effect on CTh. Additionally, no correlation was found between tumour volume or maximum diameter and CTh in the CP group. The correlations, or lack thereof, between individual clinical parameters and CTh are presented in Table 3. No statistically significant difference in CTh was found between the right and left eyes at any of the measured locations.

**Table 3:** Factors influencing choroidal thickness in the CP group.

Category	Factor	Effect on CTh	p-value
General factors	Gender (female vs. male)	No statistically significant difference in CTh; higher temporal CTh observed in females	ns
	Place of birth (urban vs. rural)	Greater SFCTh in city-born patients	<0.005
	Age at examination	No effect on CTh	ns
Tumour size	Tumour volume	No effect on CTh	ns
	Tumour diameter	No effect on CTh	ns
Tumour structure	Presence of Rosenthal fibers	No significant effect on CTh	ns
	Tumour calcification	No significant effect on CTh	ns
	Tumour morphology	No significant effect on CTh	ns
Tumour location	Cavernous sinus infiltration	Increased nasal and temporal CTh	<0.005
	Third ventricle invasion	No effect on CTh	ns
	Tumour location	No effect on CTh	ns
Complications and treatment	Hydrocephalus	No effect on CTh	ns
	VP shunt	No effect on CTh	ns

Factors influencing choroidal thickness (CTh) in pediatric patients with craniopharyngioma (CP). The table summarizes clinical and radiological parameters significantly associated with choroidal thickness in specific regions. Statistically significant differences were observed for gender, place of birth, presence of tumor calcifications, Rosenthal fibers, and cavernous sinus infiltration. CTh, choroidal thickness; CP, craniopharyngioma; SFCTh, subfoveal choroidal thickness; ns, not significant; VP, ventriculoperitoneal shunt.

## Discussion

Amid many studies evaluating CTh in systemic diseases, tumors, and vascular lesions that are accompanied by ocular symptoms, none have focused on patients with CP. To our knowledge, this is the first study to assess the impact of individual CP tumor parameters on CTh in children with CP following neurosurgical treatment, compared to healthy controls, thereby highlighting the novelty and clinical relevance of our findings.

The choroid, composed of terminal small-caliber vascular branches without collateral anastomoses (13, 30), is highly vulnerable to vascular dysfunction, which may result in local occlusion, manifesting itself as subsequent tissue thinning (31). The ability to rapidly and noninvasively image the choroid with OCT has opened new possibilities for the quantitative evaluation of CTh, providing valuable insights into choroidal status that may indirectly reflect the state of choroidal circulation. CTh measurements using OCT are noninvasive, quick to perform, and painless, and show high repeatability in the case of various devices (32, 33).

In patients with CP, tumor growth occurs in the central nervous system (CNS), compressing the optic chiasm and surrounding structures (3). This compression may impair microcirculation not only in vessels adjacent to the tumor but also in distant regions of the CNS. Such vascular changes may be associated with alterations in autoregulatory mechanisms and endothelial function, potentially influenced by chronic mechanical stress and inflammation. Importantly, our OCT measurement was conducted after neurosurgical treatment, which can directly affect blood flow in the brain and adjacent vessels, thereby influencing circulation (34). Elliott et al. (35) reported fusiform dilatation of the internal carotid artery after surgery for suprasellar tumors, particularly CP, which occurred in 10% of the operated patients. While, Macdonald et al. (36) described cerebral vasospasm with infarction after transcranial CP resection. Similarly, Madorski et al. (37), using transcranial duplex ultrasonography in 294 patients with tumors of the hypothalamic-pituitary region, found cerebral vasospasm of varying severity, which occurred in up to 62% of cases after surgical removal of tumors in the area of the sella. However, Shah et al. (38) described a case of acute spontaneous basilar artery thrombosis and posterior circulation stroke 6 days after surgery for CP.

The choroid is supplied mainly by the posterior ciliary arteries, branches of the ophthalmic artery, with venous drainage through the vortex veins into the cavernous sinus (39, 40). Postoperative changes in cerebral hemodynamics and intracranial pressure may influence choroidal microcirculation, while the effects of central nervous system alterations remain under investigation.

In our study, patients with CP were noted to have statistically significantly thinner CThs in all locations except superior to the fovea. It cannot be excluded that vascular changes, both in vessels supplying the eye and in more distant regions, could potentially affect ocular blood flow and choroidal structure. However, the extent to which these factors contribute to the observed reduction in CTh in our CP cohort remains uncertain. These results are in line with previous reports of choroidal thinning in systemic circulatory disorders, in which the primary pathology occurs distant from the eye. Several studies have documented a thinner choroid in systemic diseases, suggesting that pathological processes remote from the eye can adversely affect choroidal microcirculation (17, 41). Specifically, a thinner choroidal vascular layer has been reported in patients with kidney disease (27, 42), heart disease (25, 43), hypertension (26, 44), diabetes mellitus (45, 46), neurodegenerative disease (47, 48), and psychiatric disorder (49, 50). In contrast, our findings differ from those of Altun et al. (43), who reported increased CTh in patients with pituitary tumors based on OCT.

In our study, no statistically significant difference in CTh 1500 µm superior to the fovea was found between the CP and control groups. The greatest CTh in the CP group was located 1500 µm superior to the fovea, whereas in the control group it was subfoveal. In both groups, the thinnest CTh was observed nasally to the fovea. The CTh distribution in the control group is consistent with previous reports (32, 51). This may be due to the high metabolic demand (52) and anatomy of the eyeball, as the optic disc is the site where the optic nerve exits the eye, and the ciliary arteries pierce the sclera to supply the choroid. As a result, the nasal peripapillary region has a thicker retina and thinner choroid (51). However, this distribution, with the highest CTh observed 1500 µm superior to the fovea in the CP group, is inconsistent with previous studies and with the classical topographic pattern of CTh. This may explain the lack of statistical significance between the groups for CTh only in superior locations. This may be attributable to regional hemodynamic differences linked to compensatory mechanisms that remain poorly understood. Alternatively, this thickness pattern could reflect localized blood stasis, although a definitive explanation cannot be provided at the current level of knowledge. The study did not analyze other factors known to influence CTh, such as axial length, hormonal levels, or systemic health parameters, as these were not fully assessed and should be considered when interpreting the results.

Using OCT, Lee et al. (53) demonstrated that patients with optic chiasm compression due to a brain tumour had a lower average vessel density in the superficial peripapillary plexus in the macula of the retina and average vessel density of the radial peripapillary capillary. This confirms the microvascular changes that occur in the eyeball in tumours located in the sellar region of the CNS at a distant site, such as the eye. Other studies confirming changes in the density of peripapillary vessels in patients with chiasmal compression (54–56) and changes in the density of vessels in the choroid in patients with CP are available (57). If microvascular changes are present in the retina, both in the macula and around the optic nerve, similar alterations in the choroid cannot be ruled out. However, the authors did not analyze tumor characteristics such as size, diameter, or location, nor their potential impact on vascular changes. This makes our study the first to provide such data.

No significant correlations were found between tumor volume, diameter, or location and CTh, suggesting that choroidal microvascular alterations are complex and not directly proportional to tumor size or extent. In contrast, specific histopathological features such as tumor calcifications and Rosenthal fibers were associated with localized thinning, while cavernous sinus infiltration correlated with increased CTh at several locations. These findings should be interpreted with considerable caution due to the relatively small sample size, particularly within subgroups.

Although no statistically significant gender-related differences in CTh were found, females showed higher temporal CTh than males at a clinically relevant level. Most studies report greater CTh in men compared to women, which is thought to be related to hormonal influences and higher sympathetic nervous system activity (58, 59). However, other authors based on research on the pediatric population did not observe any correlation between CTh and sex (51, 60). Interestingly, Mapelli et al. (61) found that choroidal volume was larger in girls than in boys, contrary to some adult studies. Our study may not have sufficient power to detect an actual gender-related difference in CTh. Moreover, since our cohort consisted exclusively of children, hormonal differences might not yet be pronounced enough to affect choroidal thickness between sexes.

Our data showed no correlation between CTh and patient age within the pediatric cohort in either group. Most previous studies have reported a thicker choroid in younger children, with progressive thinning occurring with age (10, 60, 62). After age 30, the choroid typically thins by 16–20 μm per decade (52). Barteselli et al. (59) reported that choroidal volume decreases by approximately 0.54 mm^3^ per decade, corresponding to a 7.32% reduction. This finding has been previously confirmed by histological studies, which reported similar results (40, 63). Thus, our findings support the notion that age is not a significant modifier of CTh in pediatric populations. Our study failed to demonstrate a correlation between CTh and age because our group was relatively young and had a narrow age range, which would suggest a previous finding that there is no age-related reduction in CTh in the first or second decade of life.

This study has several limitations that should be acknowledged when interpreting the results. First, the relatively small sample size reflects the rarity of pediatric CP and limits the statistical power to detect subtle associations. This limitation is particularly important in subgroup analyses, where small group sizes substantially reduce statistical power and make the results more difficult to interpret with confidence. Second, the cross-sectional design precludes causal inference and does not allow for evaluation of longitudinal changes in choroidal thickness over time or in relation to disease progression and treatment effects. Third, all OCT measurements were performed after neurosurgical intervention; therefore, the direct impact of the tumor itself on choroidal thickness cannot be clearly distinguished from postoperative alterations in cerebral and ocular hemodynamics. Furthermore, based on our analysis, it was not possible to determine the relative contribution of individual tumor-related factors, including tumor location, to choroidal thinning. Additionally, multiple comparisons were performed across several parameters without formal adjustment, which may increase the risk of false-positive findings and should be considered when interpreting the results. These limitations highlight the need for larger, longitudinal, multicenter studies incorporating pre- and postoperative imaging and advanced vascular assessment to better elucidate the mechanisms underlying choroidal changes in pediatric CP.

## Conclusion

Choroidal thickness should therefore be interpreted as a non-specific marker of systemic and neurovascular alterations rather than a direct surrogate of tumor burden.

This study provides new insight into ocular microvascular alterations in pediatric CP, revealing significant choroidal thinning that likely reflects a combination of tumor effects, surgical intervention, and vascular dysregulation. OCT offers a non-invasive, reproducible modality for assessing these changes, with potential clinical utility for monitoring disease impact and guiding ophthalmologic management. Further molecular and longitudinal research is needed to dissect the pathways leading to choroidal alterations and to evaluate their prognostic significance for visual outcomes in this vulnerable population.

This manuscript was developed with support from the Medical Research Agency (ABM) as part of an educational program. It is a product of the Polish Clinical Scholar Training, organized in collaboration with Harvard Medical School's Postgraduate Medical Education. The content of this manuscript represents the author's work and is not affiliated with or endorsed by Harvard Medical School.

## Data Availability

The original contributions presented in the study are included in the article/Supplementary Material, further inquiries can be directed to the corresponding author.
